# Silver Enhances Hematite Nanoparticles Based Ethanol Sensor Response and Selectivity at Room Temperature

**DOI:** 10.3390/s21020440

**Published:** 2021-01-09

**Authors:** Daniel Garcia-Osorio, Pilar Hidalgo-Falla, Henrique E. M. Peres, Josue M. Gonçalves, Koiti Araki, Sergi Garcia-Segura, Gino Picasso

**Affiliations:** 1 Laboratory of Physical Chemistry Research, Faculty of Sciences, National University of Engineering, Av. Tupac Amaru 210, 15333 Lima, Peru; daniel.garcia.o@uni.pe (D.G.-O.); gpicasso@uni.edu.pe (G.P.); 2 Laboratory of Nanotechnology, Faculty of Gama, Area Especial de Indústria Projeção A, UNB—DF-480, University of Brasilia, Gama Leste, 72444-240 Brasilia, Brazil; pilar@unb.br; 3 Department of Electronic System Engineering, EPUSP, University of São Paulo, Av. Prof. Luciano Gualberto, 158, Trav. 3, 05508-900 São Paulo, Brazil; hperes@lme.usp.br; 4 Department of Fundamental Chemistry, Institute of Chemistry, University of São Paulo, Av. Prof. Lineu Prestes 748, Butanta, 05508-000 São Paulo, Brazil; josuemartins@usp.br (J.M.G.); koiaraki@iq.usp.br (K.A.); 5 Nanosystems Engineering Research Center for Nanotechnology-Enabled Water Treatment, School of Sustainable Engineering and the Built Environment, Arizona State University, Tempe, AZ 85287-3005, USA

**Keywords:** nano-enabled gas sensors, silver decorated hematite, alcohol sensor, hematite (α-Fe_2_O_3_), metal oxide gas sensors

## Abstract

Gas sensors are fundamental for continuous online monitoring of volatile organic compounds. Gas sensors based on semiconductor materials have demonstrated to be highly competitive, but are generally made of expensive materials and operate at high temperatures, which are drawbacks of these technologies. Herein is described a novel ethanol sensor for room temperature (25 °C) measurements based on hematite (α‑Fe_2_O_3_)/silver nanoparticles. The AgNPs were shown to increase the oxide semiconductor charge carrier density, but especially to enhance the ethanol adsorption rate boosting the selectivity and sensitivity, thus allowing quantification of ethanol vapor in 2–35 mg L^−1^ range with an excellent linear relationship. In addition, the α-Fe_2_O_3_/Ag 3.0 wt% nanocomposite is cheap, and easy to make and process, imparting high perspectives for real applications in breath analyzers and/or sensors in food and beverage industries. This work contributes to the advance of gas sensing at ambient temperature as a competitive alternative for quantification of conventional volatile organic compounds.

## 1. Introduction

The sensing of alcohol vapor enables in situ analysis and online monitoring, which allows for faster response in case of public security [1,2,3], safety risks associated with hazardous compounds [4,5,6], and food analysis [7,8,9,10]. Conventional techniques for alcohol identification and quantification such as gas chromatography and spectrophotometric analyses (i.e., IR, UV-Vis) require sample pre-treatment and sophisticated instrumentation [11,12]. Metal oxide semiconductor (MOS) based gas sensors working under the Taguchi principle [13] and are an alternative sensing technology that is not limited to online monitoring and real time analysis of analytes, in contrast with conventional analytical techniques [14,15,16,17]. Specific surface chemical transformation of MOS exposed to volatile species produces a shift of the surface oxygen reaction equilibrium state due to the presence of the target analyte. Such interactions result in a change in the amount of oxygen molecules chemisorbed on the surface, inducing a change in the resistance of the sensor material acting as a transducer. Thus, the change on the semiconductor resistance becomes the physico-chemical response related to analyte concentration [18,19].

Quantifying ethanol vapor can be required for workplace safety and health since ethanol can affect the respiratory system and is an irritant when in concentrations larger than 1000 mg L^−1^ in the gas phase [20]. Ethanol sensing is of commercial interest for the food and beverage industry since it enables online quality monitoring, allowing the evaluation of the alcohol content of drinks [21]. Additionally, consumers and governments can require ethanol sensors for noninvasive food quality control [7]. Furthermore, breath analyzers have high market value given the continuous pursuit for a low-cost and comfortable tool to estimate blood alcohol content from a breath sample [22].

The main drawback of conventional thin-layer MOS-based sensors is their limited area/volume ratio [23,24]. The small exposed area limits the sensitivity and may result in long response times, thus delaying the response recovery time. These drawbacks to full sensor competitiveness can be overcome using nano-enabled sensing surfaces [25,26,27]. Nano-structured materials can have outstanding area/volume ratio enhancing the charge transport capacity. The exploitation of this unique nano-effect may result in the development of sensors with higher sensitivity and shorter response times. Other major limitations to increase the technology readiness level and competitiveness of gas sensors are (i) the high cost of certain semiconductor materials [28,29,30] and (ii) the requirement of high temperatures for operation [31,32,33,34].

Previous works have shown outstanding performance of hematite as an ethanol gas sensor. Jia et al. prepared nanoparticles of hematite whose gas response towards ethanol was 5.54 to 50 mg L^−1^ of ethanol vapor at 240 °C, also, the authors reported Ag/α-Fe_2_O_3_ urchin-like microspheres with specific area of 20 m^2^ g^−1^ and the gas response increased up to 11.82 to 50 mg L^−1^ at 240 °C [23]. Mirzaei et al. obtained Ag@α-Fe_2_O_3_ core shell nanocomposites based gas sensors and the relative response at 250 °C was 6.0 for 100 mg L^−1^ [35]. Sensors of n-type Fe_2_O_3_ nanobelts assembled by Fan et al. showed a 2.2 gas response when exposed to 50 mg L^−1^ of ethanol at 285 °C [36]. Yan et al. prepared α-Fe_2_O_3_ nanoropes (specific surface area: 18.95 m^2^ g^−1^) sensing 100 mg L^−1^ of ethanol with 10.2 of relative signal at 240 °C [37]. The materials described above operate in the range of 250–300 °C to reach optimal response towards ethanol. The works reported in the literature rely on the use of high temperatures for semiconductor sensing, which may be a drawback for certain commercial purposes. The development of sensing strategies that enable sensing at ambient temperature is still a major challenge to promote the technology readiness level of hematite-based sensing approaches.

This work explores the use of hematite-based sensors with relatively high specific surface area as an alternative. Hematite is a n-type semiconductor material based in iron oxide, a biocompatible material made of the most abundant element in the Earth, whose electric conductivity and catalytic properties can be largely improved by incorporation of silver metal nanoparticles. Accordingly, hematite and silver-decorated hematite nanoparticles were synthesized and used to assemble gas sensors demonstrating high sensitivity, selectivity, and reproducibility to ethanol at 25 °C. To our knowledge, for the first time, the prepared sensors studied in this work operate in room conditions thanks to the improved catalytic activity provided by silver nanoparticles enhancing the sensor signal response towards that analyte in contrast with possible gaseous interferants.

## 2. Materials and Methods

### 2.1. Chemicals and Synthesis of Ag-Modified Hematite Nanospheres and Gas Sensors Assembling

Silver decorated hematite nanospheres (α-Fe_2_O_3_/Ag) were synthesized following a modified chemical co-precipitation method [38], as described in our previous work [39]. Briefly, a 125 mL solution of 0.1 mmol L^−1^ of Fe(NO_3_)_3_ (Merck Co.) and AgNO_3_ (Merck Co.) ranging from 0 up to 7.4 mmol L^−1^ was added dropwise (0.5 mL min^−1^) into 250 mL of 0.3 mol L^−1^ Na_2_CO_3_ (Riedel- deHaen Co.) solution, in the presence of 0.5 g of polyethylene glycol 2000 (Merck Co.) as surfactant. The pH of the solution was maintained constant at 10.8 during the co-precipitation process by the dropwise (1.5 mL min^−1^) addition of 0.1 mol L^−1^ Na_2_CO_3_. A brownish precipitate was formed immediately upon dropwise addition of the metal precursor. The mixture was kept under continuous magnetic stirring at 80 °C for 60 min, and the precipitate aged for an additional 12 h in static conditions. The solid was separated by centrifugation, washed three times with ethanol, and dried at 80 °C for 4 h. The recovered solid was milled using an agate mortar and uniformed using stainless steel test sieve, ø 100 μm. Then, the samples were calcined at 400 °C to ensure the formation of hematite as a pure metal oxide phase decorated with silver.

### 2.2. Assembling of α-Fe_2_O_3_/Ag Gas Sensors

**Figure 1:**
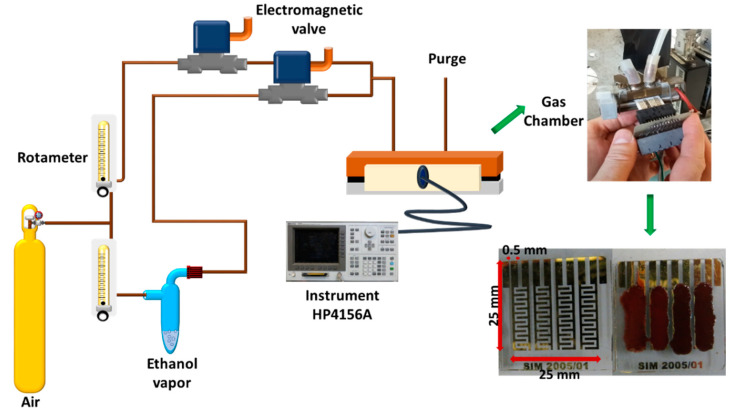
Delivery system of gases to the gas chamber with prepared sensors.

The sensors were prepared by depositing a thin layer of the nanoparticles on interdigitated gold electrodes (0.5 mm wide and 0.5 mm apart) on glass substrates (25 mm × 25 mm × 2 mm), prepared by photolithography [40]. This design was chosen to fit in printed circuit boards to connect with external instruments, see Figure 1. Previously, electrodes were cleaned with a 0.05 mol L^−1^ HCl solution, rinsed with water, and then cleaned with acetone to remove impurities and degrease the surface. The coating was deposited by screen printing using a thin brush. Briefly, a mass of 30 mg of the nanoparticles (hematite or Ag-decorated hematite) was mixed with 2 drops of ethylene glycol to form an impregnation paste. Once the paste was deposited, the screen-printed sensors were submitted to a thermal treatment at 200 °C for 8 h to remove the solvent and to stabilize the coating on the glass substrate surface.

### 2.3. Analytical Procedures and Instruments

Morphological features of nanoparticles were recorded by scanning electron microscopy (SEM) using a JEOL JSM-7401F SEM microscope (JEOL, Tokyo, Japan) at 2 kV. The samples for SEM analyses were prepared, spreading the nanoparticles powder on carbon adhesive on a copper stub. Scanning transmission electron microscopy (STEM) images were acquired with a JEOL JEM-2100F TEM-FEG microscope (JEOL, Tokyo, Japan) operating at 200 kV and micrography analyzed using the imageJ 1.53e software. Samples of as-synthesized α‑Fe_2_O_3_/Ag nanoparticles were dispersed in 3 μL of isopropyl alcohol and drop-casted onto ultrathin carbon film coated 400 mesh copper grids. Crystalline phase analysis was conducted by X-ray diffractometry (XRD) using a Bruker D2 PHASER benchtop XRD (Bruker AXS GmbH, Karlsruhe, Germany) equipped with a Cu Kα (λ = 0.15418 nm) radiation source operating at 30 kV and 15 mA. Diffractograms were recorded with a scanning window of 2θ angles of 20–70° with a 0.04° s^−1^ step size. The diffraction patterns were compared with the International Centre for Diffraction Data (ICDD) using DIFFRAC.EVA V5.2 software. Specific surface area of samples were acquired by Brunauer-Emmett-Teller (BET) adsorption analyses with N_2_ gas operating a Micromeritics Gemini VII 2390t analyzer (Micromeritics Instrumental Corporation, Georgia, GA, USA). Silver content in nanocomposite samples was quantified by inductively coupled plasma optical emission spectroscopy (ICP-OES) using an SPECTRO ARCOS ICP-OES analyzer (SPECTRO Analytical Instruments, Kleve, Germany). Samples were prepared by digesting the nanoparticle samples in 50 mL of aqua regia 8% *v*/*v* prior to analyses.

### 2.4. Sensor Testing

Sensor testing was conducted in a 10 cm^3^ gas chamber with hermetic lock in with different gas compositions feeding system for analyses, and the total flow rate of gas was 2.4 L min^−1^ that results in an average time of gas replacement in the measuring chamber of 0.25 s. The gas chamber had an inlet duct for the gas feed, and a purging duct to rinse the gas outside the chamber. The gas composition in the chamber was controlled by a dual gas delivery system consisting of (i) dry air and (ii) analyte gas. The feed of dry air was used as dilution gas to obtain working concentrations of ethanol in the range of 0 to 35 mg L^−1^. Dry air was also used as a blank and cleaning gas during hermetic chamber purge. The gas in-flow was measured with independent rotameters for each gas and controlled with electromagnetic gas valves. The composition of the gas chamber was monitored by Bruker-450 gas chromatograph (Bruker Corporation, Ontario Canada). The manufactured hematite-based sensors were put inside the gas chamber and coupled to a HP4156A Semiconductor Parameter Analyzer (Hewlett-Packard, Palo Alto, CA, USA) that is continuously registering the electrical resistance as a function of time. The test voltage was 40 V and the current through the sensors was between 9.1 × 10^−7^ to 4.8 × 10^−8^ A. Previously, all sensors were exposed to 0.5 L min^−1^ of dry air for 2 h in order to reach steady background. Then, the sensor signal (S) towards different gases was calculated according to Equation (1):

S = ΔR/R_air_ = (R_gas_ − R_air_)/R_air_, (1)

where R_air_ is the steady resistance of sensors in air, whereas, R_gas_ is measured in the presence of the analyte gas at different concentrations with a total flow rate of 2.4 L·min^−1^.

## 3. Results and Discussion

### 3.1. Hematite and Ag-Decorated Hematite Characterization

**Figure 2:**
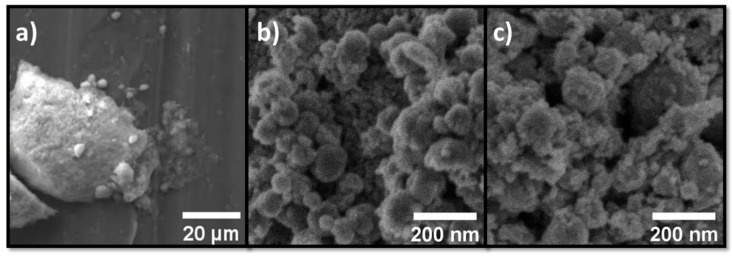
Scanning electron microscopy (SEM) micrographs of (**a**,**b**) pristine α-Fe_2_O_3_ nanoparticles and (**c**) α-Fe_2_O_3_/Ag nano-composite. Pristine hematite was obtained (**a**) in the absence and (**b**) presence of polyethylene glycol as surfactant.

The SEM images of pristine hematite and nano-composite α-Fe_2_O_3_/Ag nanoparticles are shown in Figure 2. The effect of polyethylene glycol during the precipitation of hematite can be deduced by comparing the images in Figure 2a,b. Larger agglomerates are formed in the absence of surfactant impacting the final size and homogeneity of the material. In fact, that surfactant prevents agglomeration and controls the particle size producing nanoparticles of 76 nm. The notoriously larger specific surface area of nanoparticles prepared in the presence of surfactant may benefit the sensor response. Therefore, the Ag modification was performed in the presence of surfactant. The images shown in Figure 2c illustrate that the co-precipitation method affects neither the spheroidal shape of the hematite nanoparticles nor the average size of 75 nm. Nanoparticles with similar morphology were obtained independently of the relative amount of Ag in the nanocomposite material, except for the presence of more or less large amounts of smaller silver nanoparticles (Figure 2c).

**Figure 3:**
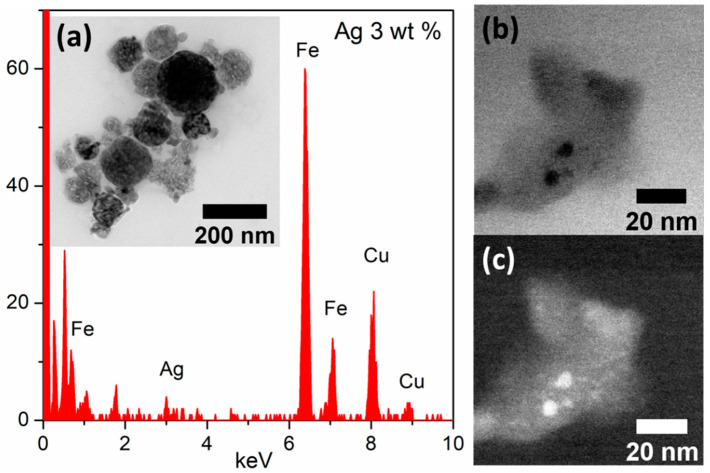
(**a**) TEM image of spheroidal nanoparticle of α-Fe_2_O_3_/Ag at 3 wt% and TEM-EDX spectra. (**b**) BF-STEM (bright-field-scanning transmission electron microscopy) and (**c**) high-angle annular dark-field (HAADF)-STEM image of α-Fe_2_O_3_/Ag at 3 wt% nanocomposite confirming the presence of AgNPs decorating the hematite nanoparticles.

The TEM micrograph of α-Fe_2_O_3_/Ag 3 wt% depicts a homogeneous distribution of approximately 76 nm diameter nanoparticles (Figure S1 in Supplementary Materials and Figure 3a). Moreover, Figure 3a revealed the chemical composition of nanocomposites by EDX analysis. As seen, iron (Fe) and oxygen (O) were the major component of the samples analyzed. Note that low quantities of silver (Ag) were observed, as expected for the low loadings of 3.0 wt% of the decorated nanocomposite. Copper signal is an artifact generated from the copper grid where the sample is supported for analysis. The bright-field (BF) STEM and high-angle annular dark-field (HAADF) images also revealed the presence of two phases, as expected for the presence of 2 to 5 nm large Ag nanoparticles decorating the hematite nanoparticles (Figure 3b,c). In fact, due to the difference in the atomic numbers between Ag and Fe, Ag nanoparticles show up as bright spots in the STEM-HAADF image (Figure 3b), and dark spots in the STEM-BF image (Figure 3c).

**Figure 4:**
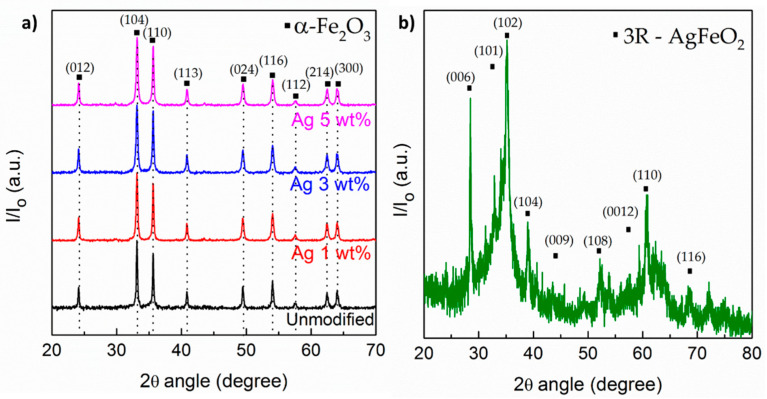
X-ray diffractogram of (**a**) pristine hematite and α-Fe_2_O_3_/Ag nanocomposites with 1 to 5.0 wt% of Ag, and (**b**) typical diffractogram of delafossite 3R-AgFeO_2_ of the composite material prepared with 10.0 wt% of silver.

Figure 4a depicts the X-ray diffractograms of hematite and nanocomposites α‑Fe_2_O_3_/Ag with silver contents of up to 5 wt%, consistent with the characteristic rhombohedral lattice system of hematite as described in [39]. Peaks associated to its typical crystallographic planes were observed at 2θ of 24.1° (012), 33° (104), 35.5° (110), 40.8° (113), 49.5° (024), 54.1° (116), 57.6° (112), 62.5° (214), and 64.1° (300), as described in the JCPDS card No. 33-0664 [41]. XRD peaks associated to silver metal were not observed in the diffractograms, which is commonly observed for nanocomposites with a high dispersion and low content of silver. However, these were clearly observed in the STEM images of Figure 3b,c that demonstrated the successful decoration of hematite NPs surface during the coprecipitation process. Conversely, when the content of Ag reaches values as high as 10 wt%, hematite is not formed during the coprecipitation process anymore. Indeed, the diffractograms of Figure 4b indicate the formation of 3R-AgFeO_2_ with delafossite structure with hexagonal lattice. Therefore, composites with more than 5 wt% of silver were excluded from our study.

The actual amounts of silver present in the hematite/silver nanocomposites were evaluated by digesting them in aqua-regia and analyzing them using a ICP-OES. As can be seen in Table 1, the silver content in the nanocomposite samples showed an excellent agreement with the iron and silver content used in the synthesis. These results, along with the STEM (scanning transmission electron microscopy) (and the enhancement of BET (Brunauer-Emmett-Teller) surface area values of Ag-hematite composites related to pure hematite (Table 1), allow us to infer that silver decorated nanoparticles were successfully prepared based on the coprecipitation method.

The current-potential curves of the assembled sensors have a linear relationship according to Ohm’s law as usually observed for ideal electric resistors (Figure S2) The smaller the slope, the higher is the resistance. Generally, materials with higher resistivity tend to have higher sensitivity for gas sensor application. Thus, the electrical responses suggest that α-Fe_2_O_3_/Ag 3.0 wt% may present higher sensitivity for gaseous analytes.

### 3.2. Comparing Hematite-Based Sensor Responses to Ethanol

**Figure 5:**
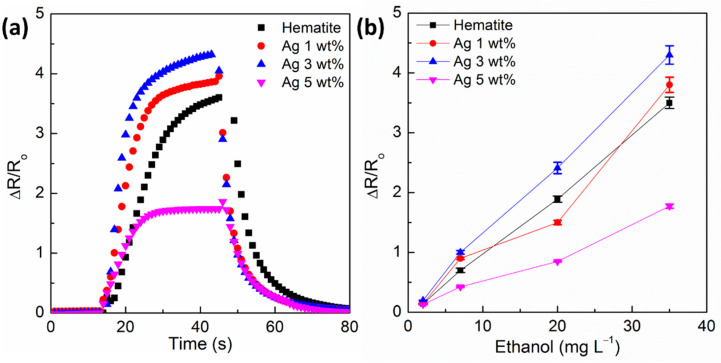
Effect of Ag loading on the sensor sensitivity to ethanol: (**a**) Comparison of the sensors signal when exposed to 35 mg L^−1^ of ethanol vapor at 25 °C, (**b**) signal response as a function of ethanol vapor concentration. Composite nanosensors: (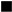) Pristine hematite, (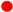) α‑Fe_2_O_3_/Ag 1.0 wt%, (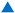) α-Fe_2_O_3_/Ag 3.0 wt%, and (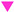) α-Fe_2_O_3_/Ag 5.0 wt%.

Sensitivity to the analyte is one of the major indicatives of sensor competitiveness. The response of the hematite-based sensors for ethanol depends on the semiconductor resistance change when exposed to the analyte according to Equation (1). Figure 5 presents the response of hematite-based sensors with different contents of silver in the nanocomposite material when exposed to 35 mg L^−1^ ethanol in gas phase, demonstrating the benefit of silver nanoparticles in the composite material. Clearly the relative signal increases in comparison to pristine hematite. Note that α-Fe_2_O_3_/Ag 3.0 wt% has about 22.9% higher signal than bare α-Fe_2_O_3_. This positive effect is explained by the role of silver in the sensing mechanism when compared to pristine hematite. Commonly, hematite as an *n*-type semiconductor must reduce its electrical resistance interacting with reducing gases, however, Figure 5 suggested that hematite behaves as a *p*-type semiconductor because the electrical resistance increased under exposition of ethanol, which is a reducing gas. This effect is not clearly described yet, but some studies attributed this behavior to low quantities of bulk impurities (e.g., Na, Mg, C, etc.) or to the annealing process in oxygen atmosphere. Both phenomena can generate holes in the lattice of hematite [42,43]. To this study, ions Na^+^ from Na_2_CO_3_ could influence the transition *n*- to *p*- of the prepared nanocomposites. Therefore, oxygen adsorbed on hematite is chemisorbed into O_2_^−^ ions (T = 25 °C) by trapping electrons from the valence band. As a consequence, a thin layer of holes in the surface of the semiconductor is formed with regards to the *p*-type behavior, and lower resistance is adopted. During ethanol release, chemisorbed O_2_^−^ and ethanol molecules react, producing CO_2,_ H_2_O, and free electrons, which return to the valence band, and the holes layer diminishes, increasing the electrical resistance of hematite [44,45]. In contrast, Ag nanoparticles (even small quantities) play a dual role as (i) electron donor and (ii) chemical sensitizer. The role of the electron donor is forming an ohmic contact at the α-Fe_2_O_3_/Ag interface, as a result of metal-semiconductor junction. Electrons transfer from the silver to hematite since the work function of hematite (φ_m_ = 5.88 eV, [46]) is higher than the silver work function (φ_m_ = 4.2 eV, [47]). Therefore, silver increases the electron density of hematite. Hence, the adsorption/desorption of oxygen molecules ions (O_2_^−^) is boosted. As chemical sensitizer, Ag promotes the adsorption of ethanol gas. Ethanol may occupy extra active sites of Ag nanoparticles, interact with oxygen ions, and enhance the electron charge transfer rate between the ethanol gas and the sensor surface. These effects make metal oxide sensors more sensitive for ethanol vapor detection, as observed in Figure 5. This higher sensitivity to ethanol concentration can be inferred from the steeper slope observed for α-Fe_2_O_3_/Ag 3.0 wt% of 0.119 L mg^−1^ than the 0.096 L mg^−1^ for pristine α-Fe_2_O_3_ (see Figure 5b).

In contrast, an excessive loading of silver has a deleterious impact and drastically decreases the sensor response. The sensor containing 5.0 wt% of Ag shows a dramatic drop in signal response down to 1.7, corresponding to a 51.4% loss in signal response in comparison to pristine α-Fe_2_O_3_, explained by the conductive character of metallic silver increasing the semiconductor conductivity while acting as charge carrier recombination sites. These effects decrease the differential resistance and interaction capacity of the surface with the analyte, thus lowering sensitivity.

**Figure 6:**
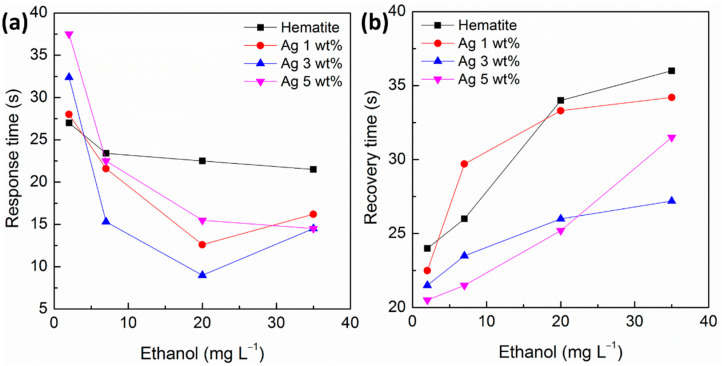
(**a**) Response time and (**b**) recovery time of the different hematite based sensors as a function of the ethanol gas concentration and nanomaterial: (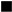) Pristine hematite, (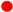) α‑Fe_2_O_3_/Ag 1.0 wt%, (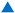) α-Fe_2_O_3_/Ag 3.0 wt%, and (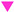) α-Fe_2_O_3_/Ag 5.0 wt%.

The response time represents the period of time taken for the sensor to react to a given stimulus. Response time is defined as 90% of the time to reach the equilibrium value of a response signal [48]. Conversely, the recovery time represents the length of time taken for the sensor to return to the initial background condition (i.e., sensor in air). By definition, the recovery time is defined as the 90% of time required to return to the original signal value when in the air after removing the target analyte gas from the measuring chamber [48]. Figure 6 presents the response time and recovery times of the α‑Fe_2_O_3_/Ag 1–5 wt% nanocomposite sensors. All the sensors showed that similar time of response is dependent on the ethanol concentration. Note that the relationship between time of response trends is dependent on analyte concentration. This is related to the diffusion velocity of the analyte and therefore should be dependent on the analyte gas concentration. Thus, shorter response times were observed for higher analyte concentrations as has been previously reported for other gas sensors operating at 25 °C [49,50]. The opposite trend is observed in Figure 6b for the recovery times, in which longer times are required for higher analyte concentrations. This is explained by the fact that during the gas purging with air, a higher number of adsorbed molecules should be displaced by oxygen.

The nanocomposite α-Fe_2_O_3_/Ag 3.0 wt% is the sensor that presents faster response and recovery times as expected by the catalytic role of Ag that enables faster adsorption/desorption of oxygen molecules on the sensor surface. All these results identify the nanocomposite with 3.0 wt% loading of Ag nanoparticles on α-Fe_2_O_3_ nanoparticle as the optimum ratio to achieve enhanced sensor response for ethanol analysis. Note that 32 s is the response time at an ethanol concentration of 2 mg L^−1^, but it is shortened to less than 15 s for concentrations larger than 5 mg L^−1^, whereas the recovery time increased from 22 to 27 s. These results evidence the rapid recovery of the sensor surface by purging with air. These short response and recovery times of α-Fe_2_O_3_/Ag 3.0 wt% nanocomposite as ethanol sensor at 25 °C are very competitive for real applications, especially the monitoring of ethanol levels in breath and in drinks.

**Figure 7:**
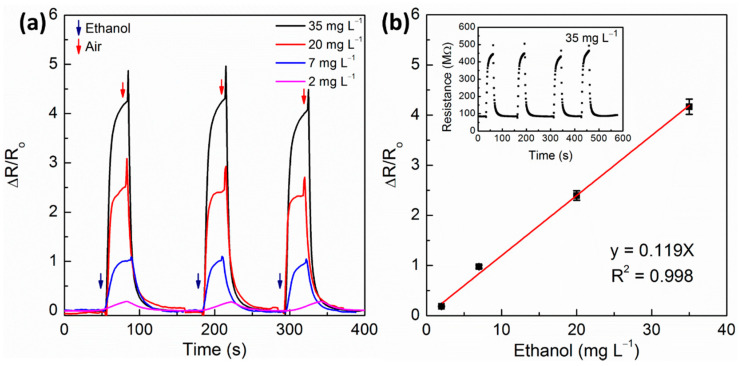
(**a**) Nanocomposite α-Fe_2_O_3_/Ag 3.0 wt% sensor response transients to consecutive cycles of exposure/purge to different ethanol vapor concentrations. (**b**) Linear relationship between concentration and relative resistance change. Inset: Electrical resistance as a function of time to 35 mg L^−1^ of ethanol.

The sensor was submitted to several cycles of analyte exposure and purging, showing excellent repeatability and stability of the response signal, as can be seen in Figure 7. Note that the relative resistance change of the sensor as response to the concentration of ethanol in the gas chamber has a good linearity and low deviation (95% confidence interval calculated to 4 values) enabling precise quantification of that analyte in different products (see Figure 7b). Besides, cyclic measurements (inset) for linear regression showed a stable baseline (R_0_) through the test. Thus, α-Fe_2_O_3_/Ag 3.0 wt% have demonstrated appropriate features for ethanol vapor sensing such as other hematite-based sensors [23,35,36,37,38]. Higher sensibility at 25 °C, short-time gas response, rapid recovery, and excellent repeatability (ΔR/R_o_), even at low concentrations (2 mg L^−1^), is the greater contribution of this work. However, relative humidity may reduce the sensor performance. Water molecules may occupy the active sites of sensors and avoid the effective detection of ethanol. High relative humidity even produces a thicker water layer on the surface and considerably reduces sensors’ activities as previously reported [51,52]. Further studies should consider the effects of variable humidities and their impact on sensitivity at ambient temperature.

**Figure 8:**
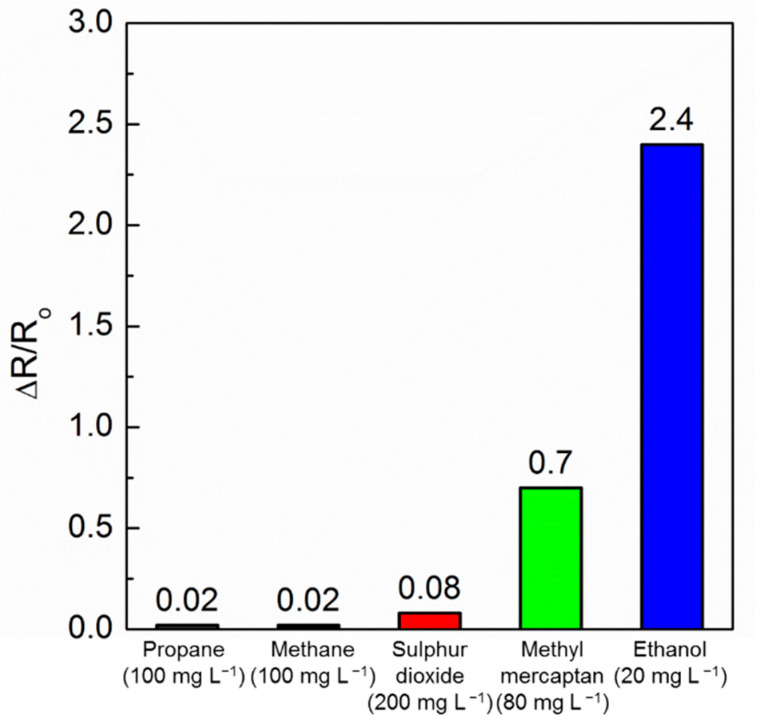
The responses of α-Fe_2_O_3_/Ag 3.0 wt% ethanol sensor at 25 °C towards possible interferant gases.

The evaluation of reactor selectivity is relevant when considering the effect of interferent species on the sensor response. The sensor based on α-Fe_2_O_3_/Ag 3.0 wt% was exposed to high concentrations of four common gas analytes (methane, propane, sulfur dioxide, and methyl mercaptan) and the impact on the sensor response depicted in Figure 8. Methane and propane at 100 mg L^−1^ showed ΔR/R_0_ signal responses as low as 0.02. Additionally, the sensor had low response to sulfur dioxide, which at concentrations of 200 mg L^−1^ gave a small signal of 0.08. The gas methyl mercaptan had a much more relevant signal with an ΔR/R_0_ response of 0.7 at 80 mg L^−1^, much lower than for ethanol. In fact, even at 4 times lower concentration of 20 mg L^−1^, the hematite-based sensor at 25 °C showed a much higher response to ethanol of ΔR/R_0_ = 2.4, which is 3.4-fold larger than that of methyl mercaptan. These results demonstrate the high selectivity of α-Fe_2_O_3_/Ag 3.0 wt% as an ethanol sensor, especially considering that methyl mercaptan will hardly be an interference in most application conditions.

## 4. Conclusions

Pristine hematite (α-Fe_2_O_3_) nanoparticle and decorated with silver were prepared according to a facile co-precipitation method and used to assemble gas sensors by depositing a thin layer on glass supported interdigitated gold electrodes by screen printing. Characterization techniques allowed inferring the successful decoration of hematite nanoparticles with Ag and the silver content quantification. The STEM images revealed the presence of 76 nm large nanoparticles whose films followed Ohm’s law. The sensor demonstrated high sensitivity towards ethanol vapor at an ambient temperature (25 °C) with good linearity in the 2–35 mg L^−1^ ethanol vapor concentration range. The results indicated that decoration of hematite nanoparticles with silver accelerates adsorption/desorption of oxygen leading to shorter sensor response and recovery times. Furthermore, the presence of silver up to 3.0 wt% enhanced the sensor sensitivity, selectivity, and reproducibility, but a larger silver loading showed a deleterious effect on sensor sensitivity as a consequence of the larger conductivity and charge recombination. Possible interferant gases such as methane, ethane, sulfur dioxide, and methyl mercaptan showed significantly lower responses as compared to ethanol, demonstrating its good selectivity. These results suggest a high potentiality of ethanol sensors based on α-Fe_2_O_3_/Ag 3.0 wt% for sensor applications in breath analyzers and/or food and beverage industries given its simple fabrication process, low cost, and sensitivity even at ambient temperature.

## Figures and Tables

**Table 1:** Elemental composition and specific surface area of hematite and α-Fe_2_O_3_/Ag nanocomposites.

Sample	(m_Ag_/m_Fe_)_theor._	(m_Ag_/m_Fe_)_exp._	BET Area/m^2^ g^−1^
α-Fe_2_O_3_	--	--	41 ± 2
α-Fe_2_O_3_/Ag 1.0 wt%	0.013	0.013	57 ± 4
α-Fe_2_O_3_/Ag 3.0 wt%	0.043	0.040	74 ± 1
α-Fe_2_O_3_/Ag 5.0 wt%	0.071	0.079	60 ± 2

## Data Availability

Data is contained within the article.

## Supplementary Materials

The following are available online at https://www.mdpi.com/1424-8220/21/2/440/s1, Figure S1: Transmission electron microscopy analysis, Figure S2: Electrical response of sensors.
