# Supplementary material for: Silver Enhances Hematite Nanoparticles Based Ethanol Sensor Response and Selectivity at Room Temperature
Source: Sensors (Basel). 2021 Jan 9;21(2):440. doi: 10.3390/s21020440 (PMC7827617; doi:10.3390/s21020440)
Supplement: Supplementary file 1 [file sensors-21-00440-s001.pdf]

Supplementary Material

Silver enhances hematite nanoparticles based ethanol sensor response and selectivity at room temperature

Daniel Garcia-Osorio, Pilar Hidalgo-Falla, Henrique E. M. Peres, Josue M. Gonçalves, Koiti Araki, Sergi Garcia-Segura and Gino Picasso-Escobar*

Corresponding author: gpicasso@uni.edu.pe

1. Transmission electron microscopy analysis

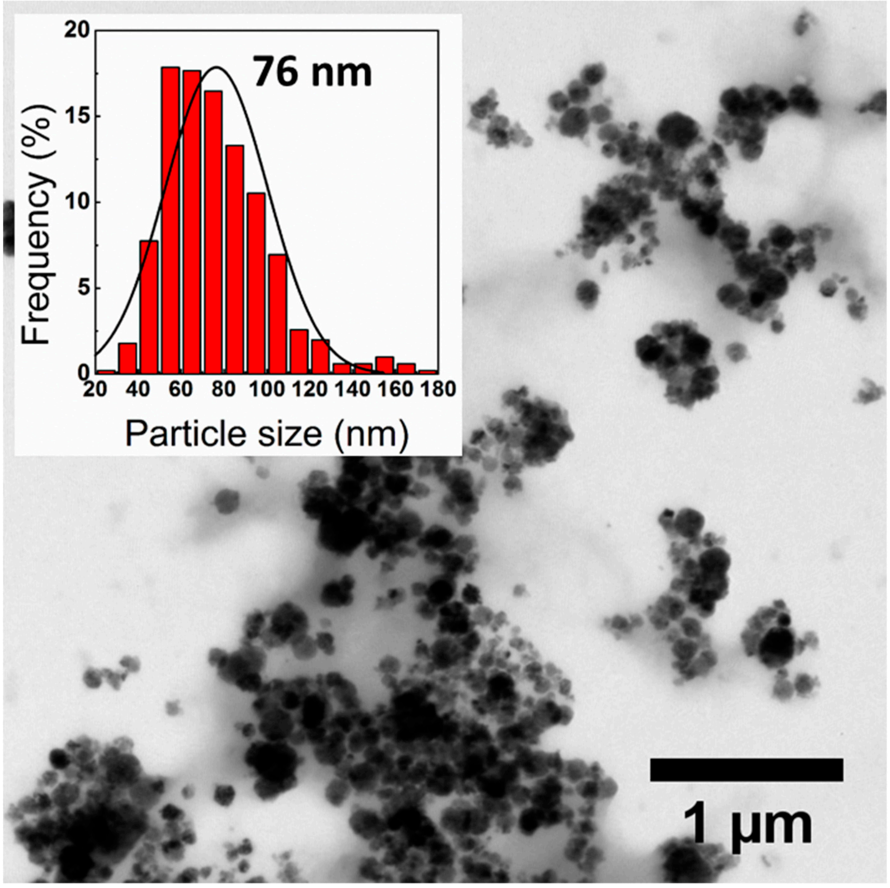

Figure S1. Particle size distribution of spheroidal nanoparticle of α-Fe2O3/Ag at 3 wt%. Data population: 500 particles.

2. Electrical response of sensors

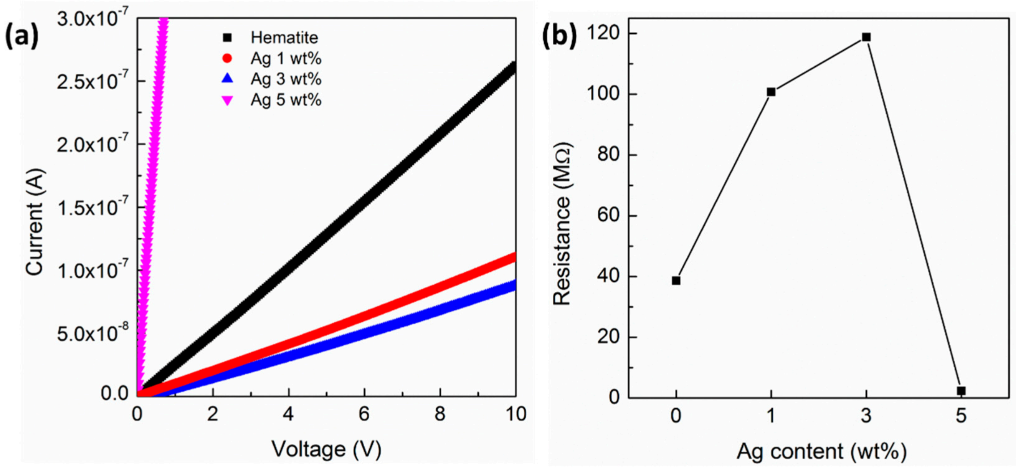

Figure S2. (a) Linear current – potential curves of prepared sensors according to the Ohm's law. (b) Resistance variation of sensors measured in air as a function of silver content.
